# Colonial signature of the alarm pheromone and chemical differences between hornet workers

**DOI:** 10.1371/journal.pone.0336261

**Published:** 2026-02-02

**Authors:** Mélissa Haouzi, Florian Bastin, Elfie Perdereau, Chloé Humbert, Bastien Play, Laureen Beaugeard, Éric Darrouzet

**Affiliations:** Institut de Recherche sur la Biologie de l’Insecte (UMR 7261) CNRS, University of Tours, Tours, France; Albert-Ludwigs-Universitat Freiburg, GERMANY

## Abstract

The social organisation of eusocial insects is based on an effective communication system in which pheromones play a central role. Among these chemical compounds, the alarm pheromone is an essential component of colonial survival by inducing nestmates recruitment and defensive behaviours. In this study, we investigated the alarm pheromone composition produced in the venom gland of workers of the invasive Yellow-legged hornet *Vespa velutina nigrithorax*, focusing on two aspects: first, variations between different colonies, and second, differences related to the activities of workers in the colony at a given time. Here, we examined four specific activities: animal foragers, builders, defenders and material foragers. Our results reveal significant chemical heterogeneity in the alarm pheromone among workers, highlighting a strong colony-specific chemical signature as well as a variability linked to workers’ activities. Notably, animal foragers and builders exhibited distinct pheromone profiles with discriminant chemical compounds. This study therefore suggests that the alarm pheromone could be used as a recognition signal in Vespidae species, both at the inter and intra-colonial levels. Such findings provide valuable insights into the chemical ecology of invasive species and open new perspectives on the role of pheromones in colony coordination and defense mechanisms.

## Introduction

In social insects, the complexity of their social structure requires precise coordination of activities among nestmates. This coordination is achieved through a diverse range of signals that ensure effective communication [1]. Chemical signals, particularly pheromones, play a central role in this communication process [2]. Alarm pheromones, a key type of chemical signal, consist of a mixture of compounds [3–5]. These pheromones are often stored in specialised gland, which can be used as a chemical reservoir to trigger social behaviour [6]. In some species, the venom gland has been identified as a key source of alarm pheromones, with the alarm signal often released alongside venom during defensive events [7–9]. A chemical compound is classified as an alarm pheromone if it is released when the colony is exposed to danger, if it is perceived by nestmates, and if it induces specific behavioral changes, such as recruitment or defense [10]. Due to their high volatility, alarm pheromones are particularly effective in mediating the rapid responses required for colonial survival.

When colonies are threatened, these volatile compounds recruit nestmates and elicit defensive behaviours [11,12]. Several alarm compounds have been identified in different species. For instance, N-3methylbutylacetamide induces attack responses in *Vespula squamosa* [13], while isopentyl acetate triggers rapid defense in honey bees [14]. Moreover, certain alarm pheromone components act synergistically, enhancing their behavioral impact. In *Vespa velutina auraria*, for example, exposure to ketones fraction from the venom gland provokes aggressive attack behaviour [9]. However, not all alarm pheromone components are highly volatile; some less volatile compounds provide longer-lasting signals. These are particularly valuable in contexts where prolonged communication is necessary. For instance, in bees, less volatile compounds serve as heterospecific alarm signals at food sources, alerting workers to potential dangers and improving foraging safety [8,15,16]. The dual functionality of volatile and less volatile compounds in the alarm pheromones mix therefore illustrates their vital role in coordinating both immediate and sustained responses within and beyond the colony.

Our study focused on the alarm pheromone of the Yellow-legged hornet, *Vespa velutina nigrithorax*. Originally from South-East Asia [17], this species was accidentally introduced into south-west France around 2004 [18]. It rapidly spread across many Western European countries [19], causing significant ecological impacts, particularly on entomofauna [20], and honey bee populations [21–23]. These disruptions have led to substantial economic losses [24,25] and public health concerns [26,27].

Despite the ecological significance of *V. v. nigrithorax*, few studies have explored alarm pheromones within the *Vespa* genus. It is known that *V. velutina* uses its sting venom as an alarm pheromone [9]. Studies on Chinese native population of *V. velutina auraria* [9], and the invasive European population of *V. velutina nigrithorax* [28] have identified some alarm compounds from the venom gland. In these studies, these compounds were tested when colonies were threatened and elicited defensive behaviours. Additional research has examined alarm pheromone composition between female castes, revealing new compounds [29–31]. However, these studies employed varying chemical extraction methods and caste analyses. Rodríguez-Flores et al. [31] analysed the *in vivo* composition of the alarm pheromone in living individuals using a headspace device, as well as the *in vitro* composition produced by the venom apparatus in workers and queens. Cappa et al. [30] analysed the composition of the venom reservoir in workers and gynes with headspace Solid Phase Micro-Extraction (SPME), while Berville et al. [29] assessed venom gland chemistry across workers, gynes, foundresses, and queens using liquid and SPME extractions. To date, a total of 35 compounds have been found in the alarm pheromone of *V. velutina* species, with evidence suggesting caste-specific variations and dynamic changes throughout the life cycle of fertile females [29]. These findings underscore the complexity and adaptability of the alarm pheromone in this invasive species.

In *V. velutina nigrithorax* workers, several distinct social behaviours have been observed, including nest maintenance, nest patrolling and foraging [32,33]. To feed larvae, *V. v. nigrithorax* acts as a generalist scavenger and insects predator, turning prey into pellets, before returning them to the colony [20,34]. More recently, four specific colonial activities have been identified, corresponding to animal foragers that bring prey pellets back to the colony; builders that construct the nest; defenders that protect the colony and material foragers that return to the nest with pellets of plant fibers mixed with saliva [33]. Among these activities, animal foragers exhibit a specific chemical signature (with cuticular hydrocarbons), suggesting a chemical task specialisation [33].

Since alarm pheromones can convey critical information to the organisation of insect societies, their composition may vary depending on factors such as the identity or role of the individuals producing them. This study aimed to analyse both the venom gland composition of workers based on (1) their colonial origin and (2) their colonial activity at a given time. We hypothesised that the alarm pheromone in Vespidae could be used as a recognition signal, enabling workers to encode and transmit distinct types of information crucial for colony coordination.

## Methods and materials

### Ethics statement

Individuals of *V. v. nigrithorax* were collected from both private properties and public spaces. For collections on private properties, consent from the owners was obtained. In public areas, permission for collection was given by local authorities responsible for treating nests. Moreover, no special authorization is necessary to keep this species individuals in laboratory conditions, or to conduct insect animal testing within the EU. The fieldwork did not involve any endangered or protected species.

### Hornet sampling

**Fig 1:**
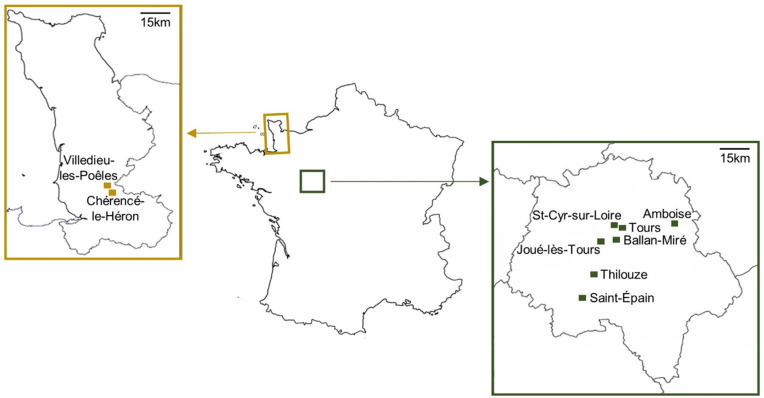
Cartography of hornets sampling in France. Nests from the department of La Manche are colored in gold, and nests from Indre et Loire are colored in green.

From mid-August to mid-October 2021, a total of 148 *V. v. nigrithorax* workers were collected from 9 colonies in France based on their observed activities (**Fig 1**). Hornet workers were identified following their activities at a given time and captured. As it was not possible to control the age of the individuals, we chose to capture only those observed outside the nest and displaying specific activities, which suggests that they were oldest adult individuals. As indicated in Haouzi et al. [33], fours type of workers were distinguished: animal foragers, builders, defenders, and material foragers. The area around the nest was cleared beforehand to ensure safe handling and to limit behavioural disturbance of the colonies. If necessary, the nest entrance was cleared of all vegetation, then left to rest for 30 minutes before any intervention. To avoid triggering defensive reactions, the hornets were captured individually using a butterfly net, approximately one meter from the nest, with the exception builders, which were gently removed using tweezers. For each type, four individuals were collected per nest, except for Amboise (animal forager, n = 5; defender, n = 3; material forager, n = 3), Ballan-Miré (animal forager, n = 6), Saint-Cyr-sur-Loire (material forager, n = 5), Saint-Épain (material forager, n = 5) and Villedieu-les-Poêles (material forager, n = 5).

### Venom gland composition analyses

Workers for each activity were freeze killed at −20°C, until they could be dissected under a binocular microscope. For each worker type, the venom gland was carefully extracted, placed in a glass vial with 200 µl of heptane and stored at −20°C. Immediately before analyses, glands were perforated manually with needle, 0.5 µl of *n*-eicosane (10^-3^ g/ml) was added to each sample as an internal standard and vials were vortexed at 300 rpm for 1 min to perform a solid-liquid extraction [29]. Two µl of each chemical sample was analysed using a gas chromatograph coupled to a mass spectrometer (GC 7890B – MS 7000C Triple Quad, Agilent Technologies, Santa Clara, CA, USA). Extracts were injected in the GC injector with a splitless time of 2 min at a constant temperature of 250°C. Helium was the carrier gas (flow rate = 1.2 ml/min) and the GC was equipped with a Zebron ZB-5HT column (30m L x 0.25 mm ID x 0.25 μm df; 5% Phenyl – 95% Dimethylpolysiloxane, Phenomenex, Torrance, CA, USA). The conditions were 70 eV electron impact ionization. The oven temperature program was used with first an initial temperature ramp from 50°C to 200°C at 8 °C/min, a second temperature ramp from 200°C to 315°C at 5°C/min, and a 5-min final hold at 315°C. Data were analysed with MassHunter Qualitative Analysis (v. 10.2, Agilent Technologies) and AMDIS v. 2.0g, NIST 2017 softwares. Compounds were identified based on fragmentation patterns of their mass spectra [35] and assigned according to previous *V. velutina nigrithorax* spectra published [29]. A similarity threshold of ≥ 80% was used for tentative identification, in combination with retention time consistency and reference to the literature when available. Peaks integration was carried out using the MassHunter Quantitative Analysis software (v. 10.2, Agilent Technologies). No manual threshold was applied to peak areas; compound inclusion was based on the software’s default integration parameters, which include internal signal detection thresholds. All matched peaks were considered regardless of their relative abundance. A solvent blank (pure heptane) was systematically injected at the beginning of each analytical run and between every group of five samples to monitor possible contamination. No significant spurious peaks were detected, and background noise remained negligible throughout the analyses.

### Statistical analyses

All statistical analyses were carried out using R software (R v4.1.0) except the LEFSe algorithm which was performed on Galaxy Community Hub. The relative abundance of chemical compounds in each chemical sample was calculated by dividing the intensity of each chromatogram peak by the sum of the peak intensities.

To assess the influence of both colonial origin and worker activity at a given time on their chemical signature, *Permutational Analysis of Variance* (*PERMANOVA*, n = 999 permutations) were performed on the Bray-Curtis dissimilarity matrices calculated from the relative abundance of chemical molecules between each pair of samples. The adonis2 function from the vegan package (v2.6-2) was used [36]. First, the interaction between the colonial origin and activity variables was tested. Because of the significant interaction identified, additional *PERMANOVA* tests were performed on the effect of colonial origin with the activity variable entered as stratification variable; and inversely for the effect of activity, where the colonial origin variable was entered as stratification variable. When significative effects were observed, *pairwise comparisons* were carried out using the pairwise.adonis2 function from the pairwiseAdonis package (v0.4) [37]. A two-dimensional Principal Coordinate Analysis (PCoA) was performed to illustrate the chemical composition dissimilarities between samples, using the ape package (v5.6-2) [38].

Then, the *Linear Discriminant Analysis Effect Size* (LEFSe) algorithm was used to identify the chemical biomarkers of animal foragers, builders and other worker types (*i.e.,* defenders and material foragers clustered and called DM). The first analysis step was a non-parametric *Kruskal-Wallis* (KW) *sum-rank test* to identify significant differences in the abundance of worker activities. Afterwards, chemical consistency was investigated using a *Wilcoxon pairwise test*. Finally, a *Linear Discriminant Analysis* (LDA) was performed to estimate the effect chemical compounds as a function of activities. Alpha values of 0.05 were performed for *Kruskal-Wallis* and *Wilcoxon tests*, and a threshold of 2 was used for logarithmic LDA scores.

## Results

**Fig 2:**
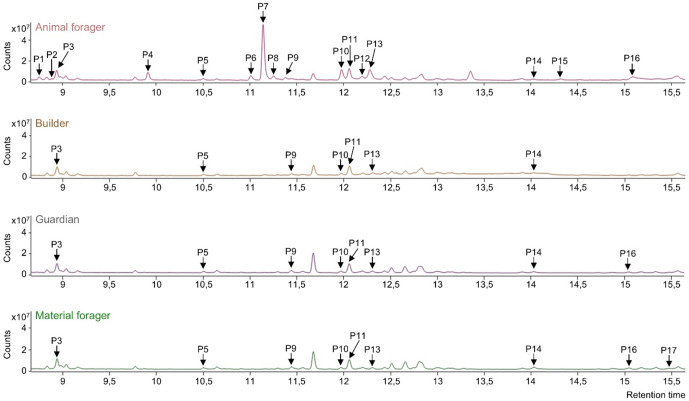
Chemical profiles of venom glands in different types of workers. The peak (P) number refers to the different compounds observed (**Table 1**). The same intensity scale was applied to all chromatograms to allow comparison.

Seventeen compounds with chain lengths ranging from 9 to 11 carbons have been analysed in chemical profiles of the venom gland volatile compounds in workers (**Fig 2**, **Table 1**). Among them, eight were ketones, four esters, two alcohols and three unknown compounds. All compounds were observed in the chemical profiles of venom glands of the different types of workers, but quantitative differences were reported.

**Table 1:** Chemical compounds from venom gland of *Vespa velutina nigrithorax* workers. The relative abundances are represented for each activity (Mean values ± Standard Deviation), *i.e.,* animal foragers, builders, defenders and material foragers.

Peak	Chemical compounds	Kovats Retention Index	Animal foragers	Builders	Defenders	Material foragers
1	Nonan-2-one	1052	5.2 ± 5.3	2.4 ± 4.3	4.1 ± 5.9	4.0 ± 5.8
2	Nonan-2-ol	1062	1.4 ± 0.8	1.0 ± 1.1	1.4 ± 1.1	1.4 ± 1.2
3	Isoamyl isovalerate	1065	9.3 ± 12.2	22.4 ± 15.7	15.6 ± 16.2	17.6 ± 19.7
4	Cyclohexanol	1117	4.4 ± 2.4	2.5 ± 2.5	3.1 ± 2.5	3.5 ± 3.2
5	X-C12:1 + 4,8-dimethyl-1,7-nonadiene	1149	1.8 ± 3.0	4.4 ± 4.3	2.9 ± 3.6	2.9 ± 4.2
6	Unknown 1	1181	2.4 ± 1.5	1.7 ± 1.9	2.0 ± 1.7	1.6 ± 2.0
7	4,8-dimethyl-7-nonen-2-one	1188	23.1 ± 12.5	10.0 ± 9.8	15.7 ± 11.6	16.9 ± 12.7
8	2-Nonanyl acetate	1195	3.0 ± 1.9	3.1 ± 3.5	3.2 ± 2.6	3.7 ± 2.9
9	β-Citronellol, methyl ether	1204	1.9 ± 1.4	0.8 ± 1.3	1.3 ± 1.3	1.3 ± 1.4
10	X’-Undecen-2-one	1234	3.24 ± 2.6	1.7 ± 2.6	2.9 ± 3.0	3.1 ± 3.0
11	X-Undecen-6-one	1242	10.7 ± 10.6	11.6 ± 6.4	9.2 ± 5.7	11.0 ± 7.0
12	Undecan-2-one	1249	6.8 ± 4.5	4.8 ± 4.4	7.2 ± 5.2	7.0 ± 4.7
13	Unknown 2	1310	13.9 ± 9.3	10.4 ± 8.9	10.2 ± 6.8	9.0 ± 6.9
14	Decan-2,9-dione + Unknown 3	1339	3.6 ± 5.5	11.5 ± 10.8	8.0 ± 8.9	5.2 ± 5.6
15	Unknown 4	1370	1.9 ± 2.0	4.3 ± 6.2	6.9 ± 17.0	6.1 ± 15.8
16	X-Undecen-2,10-dione	1416	5.6 ± 4.5	4.8 ± 4.2	4.3 ± 4.1	3.9 ± 3.9
17	Undecan-2,10-dione	1442	1.7 ± 1.9	2.5 ± 2.6	1.8 ± 2.0	1.5 ± 1.4

### Effects of colonials’ origin and activity on the venom gland composition

**Fig 3:**
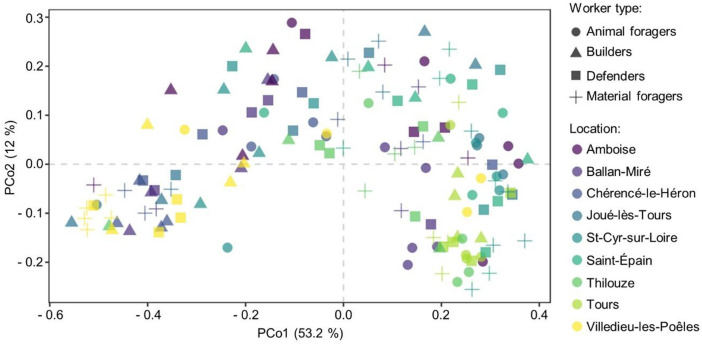
Principal Coordinate Analysis (PCoA) plot illustrating the Bray-Curtis dissimilarities between pairs of *Vespa velutina nigrithorax* chemical cues. Colonial origins are coloured and activities are represented by different logos.

The interaction between colonial origin and worker activity variables was significative (*PERMANOVA*, R² = 0.143, p = 0.029). When variables were tested independently, both colonial origin and worker activity significantly influenced the variability in the chemical compositions of the venom gland among workers and explaining respectively 30% (*PERMANOVA*, R² = 0.304, p = 0.001) and 8% (*PERMANOVA*, R² = 0.078, p = 0.001) of the variance in this chemical composition. The ordination of the different workers types in a two-dimensional PCoA showed a clear separation between some colonies, but also between animal foragers and other types of worker (**Fig 3**). Compared in pairs, animal foragers are significantly different from builders (p = 0.001), defenders (p = 0.006), and material foragers (p = 0.003). Builders are both significantly different from defenders (p = 0.009) and material foragers (p = 0.003). Conversely, defenders are not significantly different from material foragers (p = 0.759). For colonies, most of them are significantly different from each other’s (p < 0.05) (S1 Table). However, no significant differences were observed between Chérencé-le-Héron and both Villedieu-les-Poêles and Ballan-Miré, nor between St-Cyr-sur-Loire and Saint-Épain, Amboise, and Thilouze (p > 0.05).

### Chemical biomarkers between workers’ activities

**Fig 4:**
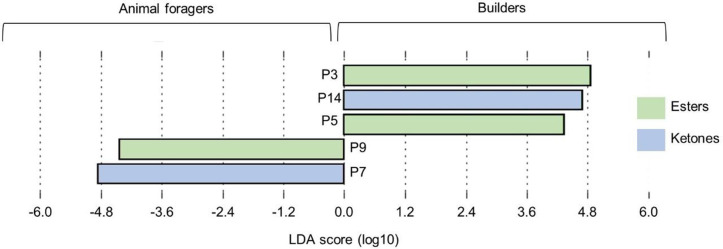
*LEfSe* representing discriminant compounds (*LDA* score (log10)>|2,5|; p < 0.05). The chemical compounds contribution at peak level is tested between animal foragers, builders, and other workers clustered (*i.e.,* defenders and material foragers, called DM).

With our model including animal foragers, builders and other workers called DM (where defenders and material foragers were clustered), five chemical compounds are significantly discriminant (*LEFSe*, < 0.05). Among them, 4,8-dimethyl-7-0nonen-2-one (P7) and β-citronellol, methyl-ether (P9) are biomarkers of animal foragers. Builders have 3 biomarkers: isoamyl isovalerate (P3), decan-2,9-dione (P14) and X-C12:1 + 4,8-dimethyl-1,7-nonadiene (P5). None chemical biomarkers has been found for DM group (**Fig 4**).

## Discussion

In this study, we demonstrate that the venom gland composition of *V. v. nigrithorax* workers varies between colonies. In ants *Oecophylla longinoda* and *Atta capiguara*, the chemical composition of mandibular glands, the organ which produced the alarm pheromone, also differs across colonies [39,40]. These cephalic odours have been shown to play a role in nestmate recognition processes in *Camponotus rufipes* ants [41]. In fact, a link between territorial behaviour and conspecific recognition has been suggested, with alarm pheromones marking territories and serving as recognition cues. Additionally, in the Vespidae species *Vespa mandarinia*, it has been shown that hornets rub their van der Vecht gland on their prey nest, emitting venom gland compounds used as recognition signals to recruit nestmates [42–44]. Our study reveals that the venom gland composition also differs between colonies in *V. v. nigrithorax*, thus supporting the hypothesis that alarm pheromones may serve as recognition cues. However, hornets from different colonies can hunt in front of the same apiary without adopting aggressive behaviour [45], which suggests that this recognition signal does not hinder the invasive success of the species.

This recognition signal is also present between populations. In fact, compared to the native population, only eight compounds have been found: nonan-2-one, nonan-2-ol, 4,8-dimethyl-7-nonen-2-one, X’-undecen-2-one, X-undecen-6-one, undecan-2-one, X-undecen-2,10-dione and undecan-2,10-dione [9]. Chemical differences between native and invasive populations could be influenced by a combination of ecological, genetic or behavioural factors, reflecting an adaptive process, but further experiments are necessary to confirm this view. For example, genetic analyses could be used to determine whether the chemical differences observed are linked to genetic divergence between native and invasive populations. In addition, behavioural tests to assess the functional role of these compounds in recognition processes, as well as the study of the impact of environmental factors on the expression of these pheromones, could provide crucial information. These approaches would provide a more detailed understanding of the mechanisms underlying the chemical differences observed between these populations.

The composition of the venom gland in workers varies depending on the activity they are performing at a given time. Except for defenders which do not differ from material foragers, all other types of workers show distinct chemical profiles. This finding is consistent with observations in other species. In honey bees, alarm pheromone production is influenced by task specialisation, with foragers producing higher level of certain chemical compounds compared to guardians, fanners or nurses [46]. Similarly, in *Apis cerana*, foragers produce more benzyl acetate than guardians [47]. The alarm pheromone composition can also be modulated by the age of individuals. In bees, 2-heptanone increases throughout the life cycle [48], while isopentyl acetate is absent in newly emerged individuals, but present in older ones [49]. In *Platythyrea punctata* ants, younger individuals performing tasks inside the colony produce lower quantities of (*S*)-(−)-actinidine and (*S*)-(−)-citronellal compared to foragers [50]. Furthermore, we cannot draw any conclusions regarding the effect of age, as our study was conducted in the field on colonies discovered by citizens. In *V. v. nigrithorax*, the presence of an age polyethism is unknown to date, but recently a task specialisation has been suggested where animal foragers exhibit a specific cuticular hydrocarbons profile [33]. Our findings therefore could support this hypothesis, but further behavioural studies are necessary to confirm this point of view. Future studies could focus on monitoring individual workers throughout their life cycle in the wild, in order to observe age-related changes in tasks. However, this approach could prove difficult, as it is currently impossible to remove young workers without risking destroying the nest, which would destabilise the social organisation of the colony.

Juvenile hormone (JH) may also play a role in modulating alarm pheromones and aggressive behaviours. In *Apis mellifera*, application of a JH analogue induces premature production of 2-heptanone and isopentyl acetate, two key alarm compounds [51]. Furthermore, in honey bees, JH levels correlate with aggressive behaviour [52]. This hormonal regulation could similarly explain the chemical specialisation observed in hornet workers performing different activities at a given time. Further studies are needed to investigate the potential relationship between JH levels in hemolymph, worker activity, and the composition of their alarm pheromones.

In this study, five key discriminant compounds were identified in animal foragers and builders. Among these, 4,8-dimethyl-7-nonen-2-one was found to be characteristic of animal foragers, and it has previously been described as a major compound in this species [29]. It is also known in *Vespa orientalis,* that ketones elicit alarm behaviour [53]. In addition, β-citronellol, methyl-ether is another compound associated with animal foragers. As these foragers hunt prey, it is possible that they use this compound as a toxic agent to kill them, although its precise role remains unknown and warrants further investigation.

In builders, isoamyl isovalerate appears to be a key chemical determinant of this activity. This compound may be common within the *Vespa* genus, as it has also been identified in the venom of *Vespa crabro* [54]. Similarly, decan-2,9-dione, previously identified but not emitted during stress [29], was also found to be a chemical marker for builders in this study. This suggests that builders, which remain close to the nest, may not require compounds typically emitted during stressful events to repel or attract individuals. Alarm pheromones are known to have a dual function in Vespidae. As in addition to activating nest defence and inducing nestmate recruitment [12], alarm pheromones also play a role in marking foraging sites, as demonstrated in *Vespa mandarinia* [42]. As previously mentioned, hornets recruit nestmates to foraging sites by rubbing their van der Vecht glands, which release venom gland compounds. Variability in alarm pheromone composition is thus crucial for hornet colony organisation. In our study, the chemical differences observed between animal foragers and builders may convey distinct information. It is possible that animal foragers use the alarm pheromone to mark foraging sites, as observed in other hornet species, while builders may use it to signal a defect or a break in the nest, indicating an urgent need for repairs. Further behavioural studies are required to explore these hypotheses.

In conclusion, this study reveals significant chemical variation in the composition of the venom glands of *V. v. nigrithorax* workers, both between colonies and according to their activity at a given time. We have identified distinct chemical markers for different types of workers, such as foragers and builders, or certain compounds appear to be linked to specific behaviours according to the scientific literature. The chemical differences observed between colonies suggest the presence of recognition processes, while the variation between worker activities could have a dual function: marking foraging sites for animal foragers and signalling nest maintenance needs for builders. These hypotheses are based on observations made in other insect species, where task specialisation influences chemical profiles, but future behavioural experiments are needed to confirm this point of view in *V. v. nigrithorax*. Furthermore, due to the limitations of our study, we cannot definitively conclude on task specialisation. It could be that hornets change tasks or perform several tasks at the same time. *V. v. nigrithorax* is a difficult biological model to maintain under laboratory conditions that faithfully reflect natural behaviour. In addition, sampling was based on citizen reports, which gave a limited data set, making it difficult to control the age of the individuals sampled or to standardise the environmental context. In addition, the lack of temporal tracking prevented us from assessing whether individuals could change task over time or correlate age with task specialisation. These factors, combined with the inherent variability of the field data, preclude drawing definitive conclusions about task-specific chemical profiles. Nevertheless, this study represents an important first step, providing valuable information on the chemical ecology of this invasive species and opening new avenues of research into the role of pheromones in the coordination of individuals within colonies and defence mechanisms. These findings may also help explain the challenges in developing an effective pheromonal trap for this species.

## Supporting information

Table S1 P-values obtained by pairwise adonis comparisons, using the Permanova tests between workers colonies. Bold values are statistically significant (p < 0.05).
(PDF)
