# Supplementary material for: Colonial signature of the alarm pheromone and chemical differences between hornet workers
Source: PLoS One. 2026 Feb 2;21(2):e0336261. doi: 10.1371/journal.pone.0336261 (PMC12863491; doi:10.1371/journal.pone.0336261)
Supplement: Table S1 — Bold values are statistically significant (p < 0.05). (PDF) [file pone.0336261.s001.pdf]

Table S1. P-values obtained by pairwise adonis comparisons, using the Permanova tests between workers colonies. Bold values are statistically significant ($p < 0.05$).

	Villedieu-les-Poêles	Ballan-Miré	Chérencé-le-Héron	Joué-lès-Tours	St-Cyr-sur-Loire	Saint-Épain	Thilouze	Tours
Ballan-Miré	**0.001**	-	-	-	-	-	-	-
Chérencé-le-Héron	0.103	0.059	-	-	-	-	-	-
Joué-lès-Tours	**0.006**	**0.024**	**0.016**	-	-	-	-	-
St-Cyr-sur-Loire	**0.001**	**0.001**	**0.001**		-	-	-	-
Saint-Épain	**0.001**	**0.001**	**0.001**	**0.002**	0.087	-	-	-
Thilouze	**0.001**	**0.034**	**0.001**	**0.005**	0.065	-	-	-
Tours	**0.001**	**0.001**	**0.001**	**0.001**	**0.001**	**0.001**	**0.001**	-
Amboise	**0.001**	**0.034**	**0.001**	0.084	**0.037**	**0.006**	**0.023**	**0.001**
